# Supplementary material for: In Vitro Characterization of the Physical Interactions between the Long Noncoding RNA TERRA and the Telomeric Proteins TRF1 and TRF2
Source: Int J Mol Sci. 2022 Sep 9;23(18):10463. doi: 10.3390/ijms231810463 (PMC9500956; doi:10.3390/ijms231810463)
Supplement: Supplementary file 1 [file ijms-23-10463-s001.zip › ijms-1880340-supplementary.pdf]

Supplementary Material for

***In vitro* characterization of the physical interactions between the long noncoding RNA TERRA and the telomeric proteins TRF1 and TRF2**

Patricia L. Abreu, Yong Woo Lee, Claus M. Azzalin

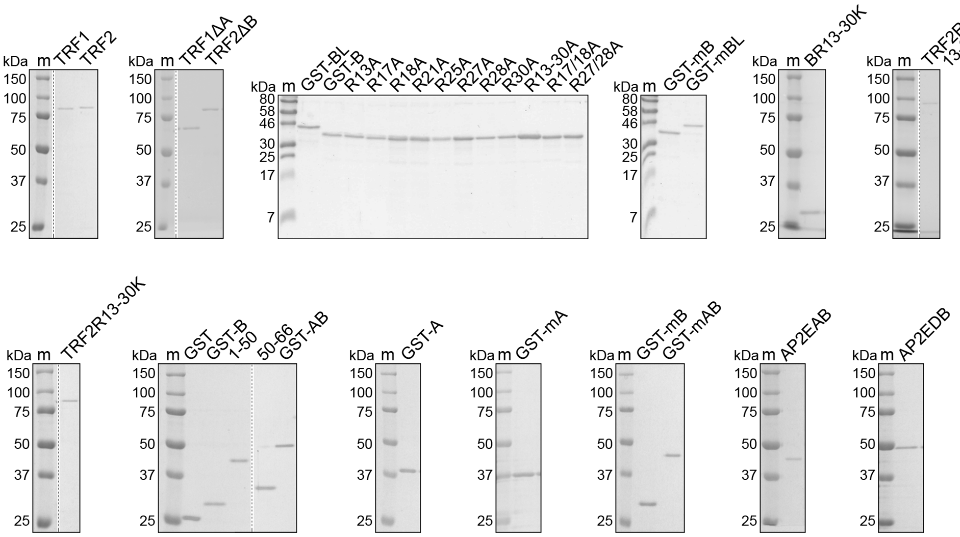

Supplementary Figure S1. Recombinant proteins used in this study. Recombinant proteins were size-fractionated by SDS-PAGE and stained with BlueSafe reagent. Molecular weights of size markers (m) are on the left of each gel in kDa.

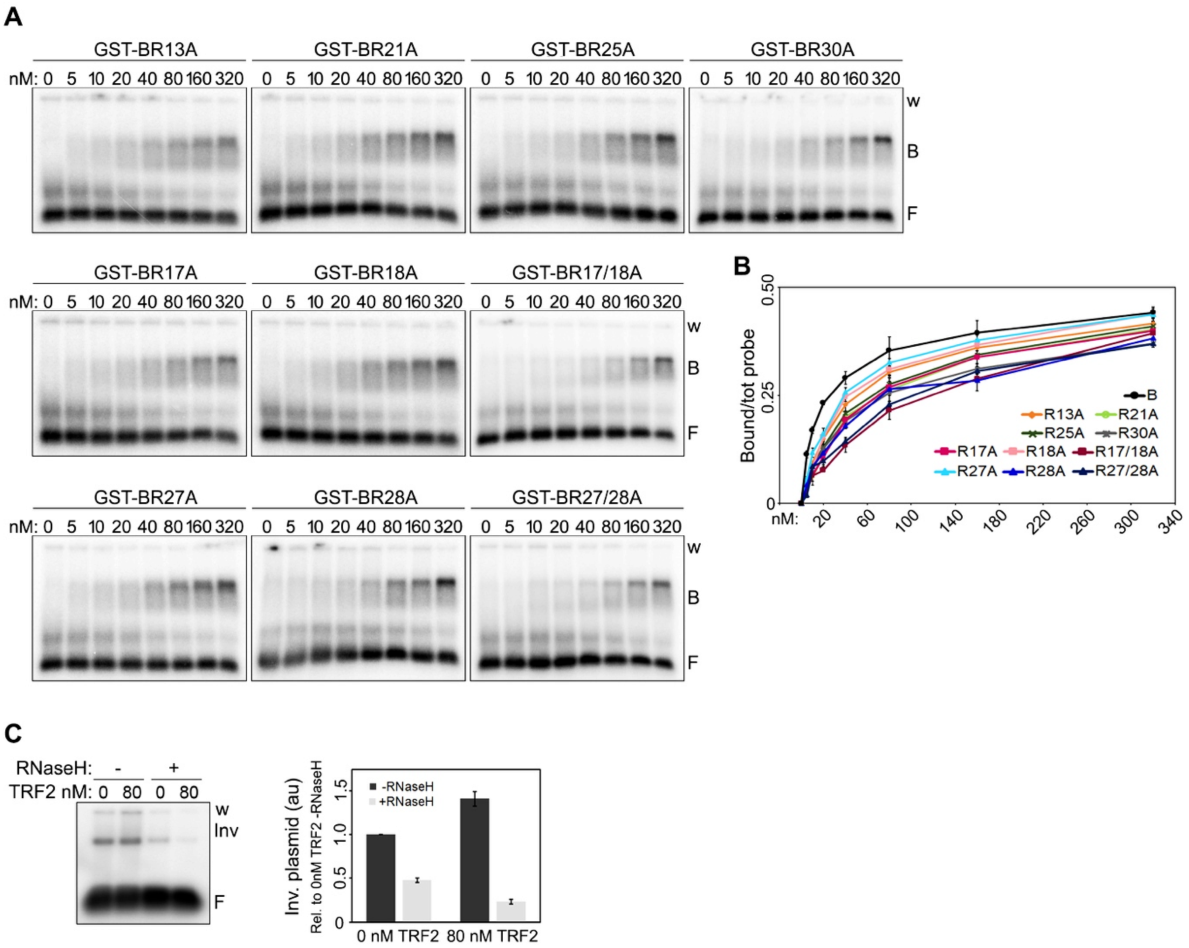

Supplementary Figure S2. Binding of human B substitution mutants to TERRA (related to Figure 3) and RNAseH controls for plasmid invasion assays (related to Figure 4). **(A)** EMSAs with radiolabeled (UUAGGG)5 TERRA oligonucleotides (0.25 nM) and increasing amounts of the indicated recombinant proteins. GST, glutathione S-transferase; B, bound probe; F, free probe; w, wells. **(B)** Bound oligonucleotides were quantified and graphed as fraction of the total signal within each lane. Data points are means and error bars are SDs (n=3 independent experiments except for GST-B18A and GST-B28A for which n=2). **(C)** A plasmid containing a 1.2 kb long telomeric repeat sequence (0.15 nM) was incubated with radiolabeled (UUAGGG)5 TERRA oligonucleotides (1 nM) in the presence or absence of TRF2 (80 nM) and of RNaseH (60U). Inv, invaded plasmid; F, free probe; w, wells. Invaded plasmids were quantified as the fold difference compared with sample lacking TRF2 and RNaseH. Bars are means and error bars are SDs (n=2 independent experiments).
